# Plasma amino acid profiles are associated with insulin, C-peptide and adiponectin levels in type 2 diabetic patients

**DOI:** 10.1038/nutd.2014.32

**Published:** 2014-09-01

**Authors:** H Nakamura, H Jinzu, K Nagao, Y Noguchi, N Shimba, H Miyano, T Watanabe, K Iseki

**Affiliations:** 1 Institute for Innovation, Ajinomoto Co., Inc., Kawasaki, Japan; 2 R&D Planning Department, Ajinomoto Co., Inc., Tokyo, Japan; 3 Link and Communication Co., Inc., Tokyo, Japan; 4 Iseki Internal Medicine, Tokyo, Japan

## Abstract

**Objectives:**

Plasma-free amino acid (PFAA) profiles have been associated with a future risk of developing diabetes or cardiovascular disease in nondiabetic subjects. These PFAA alterations might predominantly result from the metabolic shift caused by insulin resistance and visceral fat deposition. The variety of PFAA profiles within diabetic subjects is not well researched. In this study, we focused on type 2 diabetic subjects and examined the association between PFAA profiles and insulin- and glucose-related variables.

**Methods:**

Fifty-one Japanese subjects diagnosed with type 2 diabetes were recruited from an outpatient clinic. The plasma concentrations of 21 amino acids; glucose-related markers including glucose, hemoglobin A1c (HbA1c), glycoalbumin and 1,5-anhydroglucitol; insulin-related markers including insulin, C-peptide, and the homeostasis model assessment of insulin resistance; and adipocytokines including adiponectin and leptin were determined. The association of PFAA and other metabolic profiles were analyzed, and stratified analyses of the PFAAs and clinical characteristics were performed according to the fasting plasma insulin and HbA1c levels. In addition, the PFAA indices that correlate to visceral fat obesity were evaluated.

**Results:**

Although strong correlations between PFAAs and glucose-related markers were not observed, several amino acids (branched-chain amino acids, tryptophan, alanine, tyrosine, glutamate and proline) and PFAA indices that evaluate visceral obesity were highly correlated with insulin-related markers and adiponectin (*P*<0.001). In the group of diabetic patients with hyperinsulinemia, the amino acid levels were significantly increased, which generally demonstrated good concordance with insulin-related markers and adiponectin levels.

**Conclusions:**

The PFAA profiles in diabetic patients were strongly associated with hyperinsulinemia and hypoadiponectinemia, which might become risk evaluation factors for the development of cardiovascular diseases.

## Introduction

Several recent reports showed that the plasma-free amino acid (PFAA) profiles are altered before the development of type 2 diabetes or cardiovascular events. The Framingham Offspring Study recently revealed that the PFAA levels, particularly the levels of branched-chain amino acids (BCAAs) and aromatic amino acids, were significantly associated with a future diagnosis of diabetes mellitus^1^ and cardiovascular diseases.^2^ We previously revealed that several amino acids were significantly altered in Japanese people with high visceral obesity independent of their body mass index (BMI).^3^ In addition, a specific formula incorporating six amino acid values (alanine (Ala), glycine (Gly), glutamate (Glu), tryptophan (Trp), tyrosine (Tyr), and BCAA) was developed for discrimination of subjects with high visceral fat accumulation by multivariate logistic regression analyses, and this formula was strongly correlated with visceral fat deposition independent of the BMI. Because hyperinsulinemia and the development of type 2 diabetes are strongly associated with visceral obesity, diabetic patients are expected to show different plasma amino acid patterns and a higher value of this index. Although the plasma concentrations of some amino acids have been reported to be altered in diabetic patients compared with those of healthy individuals,^4^ the relationships between PFAA concentrations and the index value or other biochemical markers such as glucose- and insulin-related variables, particularly within subjects with type 2 diabetes, remain unclear.

Adipocytokines are key modulators of insulin sensitivity that carry information regarding visceral and subcutaneous fat deposition to other tissues.^5,6^ Adiponectin is known as an adipose tissue-derived insulin sensitizer, and it modifies glucose homeostasis and exhibits anti-inflammatory and anti-atherogenic effects.^7^ Insulin resistance is a major pathophysiological factor of diabetes, and adiponectin, given its strong association with insulin sensitivity and its underlying mechanism to sensitize insulin, is hypothesized to be centrally involved in the events leading to diabetes.^7^ This finding is supported by extensive epidemiological data^8,9^ linking lower adiponectin levels to disease states including type 2 diabetes, metabolic syndrome, hypertension, cardiovascular disease and cancer. Another adipocytokine, leptin, is a protein that circulates in proportion with the body fat mass and provides information regarding the nutritional status and subcutaneous fat mass to neural centers that regulate feeding behavior, appetite and energy expenditure.^6^ In addition, leptin levels are reported to be an independent risk factor for coronary heart disease.^10^ Although many studies show the relationship between these adipocytokines and the development of diabetes, research examining the relationships between plasma amino acid concentrations and adipocytokines in diabetic subjects are limited.

In this study, we measured glucose- and insulin-related variables and plasma amino acid concentrations in people diagnosed with type 2 diabetes. The PFAA concentrations and amino acid index values that evaluate visceral fat accumulation were analyzed according to their fasting plasma insulin or hemoglobin A1c (HbA1c) levels. Their correlations with adipocytokines such as adiponectin and leptin were examined. Through these analyses, we characterized the PFAA profiles and amino acid indices in type 2 diabetic patients.

## Materials and Methods

### Ethics statement

This study was conducted in accordance with the Declaration of Helsinki, and the protocol was approved by the ethics committees of Ajinomoto Co., Inc. All the subjects gave their written informed consent for inclusion before they participated in the study. All the data were analyzed anonymously throughout the study. The study was registered in the University Hospital Medical Information Network Clinical Trials Registry (UMIN-CTR) UMIN000011594.

### Subjects

This study was a cross-sectional evaluation of the relationship between the PFAA profiles and insulin- and glucose-related variables in 51 Japanese subjects who had been diagnosed with type 2 diabetes and were under treatment at the Iseki Internal Medicine. The participants were patients over the age of 20 years who have no serious health problems including cardiovascular diseases. The exclusion criteria at the beginning of the study were pregnancy, mental disorders and cancer. Hypertension and dyslipidemia occurred with type 2 diabetes in 37% (19/51) and 39% (20/51) of the subjects, respectively. According to the criteria, obesity was defined as a BMI of 30 kg m^−^^2^ or greater, and overweight was defined as a BMI of 25 kg m^−^^2^ or greater; 18% (9/51) and 55% (28/51) of the patients were categorized as obese or overweight, respectively. Seventy-one percent (36/51) of the patients were taking antidiabetic drugs at the time of the blood collection. The smoking status was not recorded.

### Analysis of metabolic variables and hormones

Blood samples (5 ml) were taken from forearm veins after overnight fasting. The serum levels of high-density lipoprotein cholesterol, low-density lipoprotein cholesterol and triglyceride were determined enzymatically. The fasting plasma glucose (FPG) levels were measured using the hexokinase method, and HbA1c was determined using the latex agglutination immunoassay. The plasma levels of glycoalbumin (GA), 1,5-anhydroglucitol (1,5-AG), insulin, C-peptide, leptin and adiponectin were measured immunologically. The homeostasis model assessment of insulin resistance (HOMA-IR) was calculated using the following equation: HOMA-IR=Fasting insulin level (μU ml^−1^) × Fasting plasma glucose level (mg dl^−1^) / 405.^11^ For evaluating insulin resistance with HOMA-IR, FPG should be less than 140 mg per day, and thus, the analysis of HOMA-IR in this study has limitations.

### PFAA measurement

The blood samples in tubes containing disodium ethylenediaminetetraacetate (Terumo, Tokyo, Japan) were immediately placed on ice. The plasma was prepared by centrifugation at 3000 r.p.m. at 4 °C for 15 min and then stored at −80 °C until analysis. The plasma samples were deproteinized with acetonitrile at a final concentration of 80% before measurement. The plasma amino acid concentrations were measured by high-performance liquid chromatography–electrospray ionization mass spectrometry followed by precolumn derivatization, as described previously.^12, 13, 14^ The following 21 amino acids were measured: Ala, alpha-aminobutyric acid (a-ABA), arginine (Arg), asparagine (Asn), citrulline (Cit), Glu, glutamine (Gln), Gly, histidine (His), isoleucine (Ile), leucine (Leu), lysine (Lys), methionine (Met), ornithine (Orn), phenylalanine (Phe), proline (Pro), serine (Ser), threonine (Thr), Trp, Tyr and valine (Val). We calculated two ‘amino acid indices' based on the PFAA levels, which correlate with visceral fat deposition by computed tomography scanning.^3^ The amino acid index-1 is the multivariate PFAA logistic regression model that discriminates subjects with high visceral fat area (⩾100 cm^2^) consisting of Ala, Gly, Glu, Trp, Tyr and BCAA, whereas the amino acid index-2 is the multiple linear regression model with variable selection to model the relationships between the PFAA profiles with the visceral fat area, consisting of Ala, Asn, Gly, Trp, Tyr and Val.^3^

### Statistical analysis

The statistical and multivariate analyses such as the cluster analysis and correlation analysis were performed with the JMP 9.0.0 program (SAS Institute Inc., Cary, NC, USA). The hierarchical cluster analysis by Ward's method was performed on the basis of the relative value of each individual variable. The data in the tables are expressed as the mean±s.d. Student's *t*-test was used to compare the variables between the high and low groups of fasting plasma insulin or HbA1c levels. Statistical significance was set at *P*<0.05. To evaluate the correlations among the variables, Pearson's product-moment correlation coefficients were calculated.

## Results

### Cluster analysis of PFAA levels and other variables

**Figure 1:**
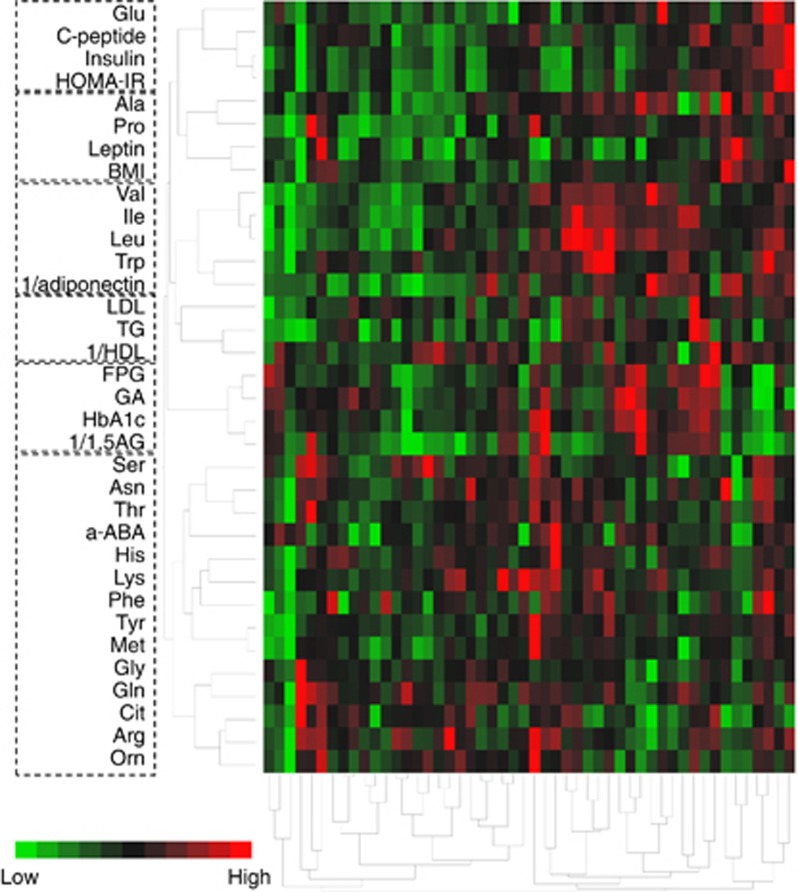
Cluster analysis of plasma amino acid and other clinical variables in type 2 diabetic patients. The dendrograms obtained from the hierarchical cluster analysis of the plasma amino acids and some clinical variables relating to glucose, insulin resistance and lipid homeostasis in 51 type 2 diabetes patients are shown. Ward's method was used for the analysis. The red block represents a relatively high level and the green block represents a relatively low level. The dotted line represents the cluster branches of the dendrogram. HDL, high-density lipoprotein; LDL, low-density lipoprotein; TG, triglyceride.

A total of 51 subjects with type 2 diabetes were enrolled in the study. To clarify the network structure between PFAA and other variables, cluster analysis was performed on the basis of the relative value of each variable in this study (Figure 1). The colored blocks represent the plasma amino acid concentrations and variables of each individual sample. Red represents a relatively high concentration, and green represents a relatively low concentration. Although the patients were all diagnosed with type 2 diabetes, their amino acid profiles were markedly different from each other, and some distinguishable clusters formed. Glu was in the same cluster as insulin-related variables such as insulin, C-peptide and HOMA-IR. In addition, Ala and Pro were categorized in the same group as leptin and BMI. Adiponectin was categorized in the cluster containing BCAAs and Trp, which was different from leptin, one of the major adipocytokines. The lipid variables (low-density lipoprotein, high-density lipoprotein cholesterol and triglyceride) and the glucose-related variables (FPG, HbA1c, GA and 1,5-AG) formed their own clusters that contained no amino acids. We could not find a specific tendency regarding the drugs used in this study.

### Characteristics of the subjects enrolled in the study

To unveil the relationship between the PFAAs and insulin or glucose-related markers, the subjects were classified into two groups according to their fasting plasma insulin concentrations or HbA1c. The fasting insulin values ranged from a minimum of 2.1 μU ml^−1^ to a maximum of 32.2 μU ml^−1^ (male: 3.1–32.2 μU ml^−1^, female: 2.1–16.1 μU ml^−1^). The HbA1c values ranged from a minimum of 5.1% to a maximum of 9.4% (male: 5.1–9.4%, female: 6.1–8.6%). The cutoff values of the insulin concentration and HbA1c between the high and low groups were 7.1 μU ml^−1^ and 7.5% in males and 9.1 μU ml^−1^ and 7.1% in females, respectively.

The age, BMI, sex and metabolic variables within each group are summarized in Table 1. Greater levels of C-peptide and HOMA-IR and lower adiponectin concentrations were observed in the hyperinsulinemia group in both sexes. Significant differences in BMI, high-density lipoprotein cholesterol, triglyceride and leptin between the high and low insulin groups were observed only in the female subjects. The group with higher HbA1c levels exhibited higher levels of FPG and GA and lower 1,5-AG levels in both sexes. These results indicate that the plasma fasting insulin concentration and HbA1c levels are independent of each other. We could not find a specific tendency of the use of antidiabetic drugs regarding the insulin levels, whereas higher ratio of taking the drugs was observed in the female group with higher HbA1c level.

### PFAA profiles according to the fasting plasma insulin and HbA1c levels

Table 2 shows each plasma amino acid concentration according to the difference between the fasting plasma insulin levels and HbA1c. The plasma levels of Tyr, BCAAs (female), Met (female), Ala (female), Pro (male) and Glu (male) were significantly increased in the hyperinsulinemia group. According to the HbA1c levels, significant differences in the levels of PFAA between the high and low groups were only detected in Ile and Leu levels in female.

The values of the amino acid indices (1 and 2) evaluating visceral fat accumulation from the PFAA profiles were much greater in the hyperinsulinemia groups in both sexes (amino acid index-1; 1.88±0.85 vs 0.59±0.87 in males, *P*<0.01, 0.79±1.09 vs −0.09±0.88 in females, *P*<0.05, amino acid index 2; 18.6±2.7 vs 15.2±3.3 in males, *P*<0.05, 17.3±3.3 vs 13.6±3.2 in females, *P*<0.01). The values were not affected by the difference in the HbA1c level in both sexes.

### Correlation between PFAA and clinical profiles

Pearson's correlation coefficients between the PFAAs and the variables related to diabetes are shown in Table 3. The glucose-related variables such as FPG and HbA1c, which are widely used for the diagnosis of diabetes, were highly correlated with the other glucose-related variables such as GA and 1,5-AG (*r*=0.778 and −0.629 for FPG, *r*=0.761 and −0.758 for HbA1c, respectively) and showed low correlations with the insulin-related variables such as C-peptide, insulin and HOMA-IR.

The Ala, Pro and Tyr concentrations were significantly correlated with the BMI. The Ser, Gln, Thr and Lys concentrations were correlated with the FPG, and the Pro and BCAA concentrations were correlated with the HbA1c. Few amino acids were correlated with GA or 1,5-AG. However, Glu, Tyr, Ala, Pro and BCAAs were strongly correlated with the insulin-related variables such as C-peptide, insulin and HOMA-IR. The plasma adiponectin levels were highly correlated with Glu, Ala, Trp and BCAAs. No amino acid was significantly correlated with leptin. In addition, the amino acid indices exhibited a higher correlation with the insulin-related variables than with the glucose-related variables. The amino acid index-1 and -2 were highly correlated with adiponectin (*r*=−0.523 and −0.468, respectively), whereas no significant correlations were found with the leptin level.

## Discussion

In this study, we demonstrated a variety of PFAA profiles in type 2 diabetic patients and reported that the PFAA profiles and amino acid indices evaluating visceral obesity showed good correlations with insulin-related variables and adiponectin concentrations.

In the network analysis with type 2 diabetic patients (Figure 1), the hierarchical cluster revealed that insulin-related variables such as plasma insulin, C-peptide and HOMA-IR were categorized into a group that was different from that of glucose-related variables such as FPG, HbA1c, GA and 1,5-AG. The concentrations of insulin-related variables did not differ between the two groups when divided by their HbA1c values (Table 1), and no significant correlations were found between insulin and FPG or HbA1c (data not shown). These data indicate that the insulin- and glucose-related variables were independent of each other, although it is generally known that hyperinsulinemia and hyperglycemia are related to each other in the process of developing diabetes. Some studies have reported that only the fasting insulin level and not the fasting glucose or HbA1c is associated with future coronary heart disease and stroke events,^15^ and insulin resistance predicted the subsequent onset of heart failure independently of established risk factors including diabetes,^16^ indicating their independent relationships. Our analysis revealed that the leptin level, and not the adiponectin level, was categorized into the identical group as the BMI, suggesting that both variables reflect different metabolic states. A previous study showed that adiponectin concentrations are more strongly influenced by the accumulation of visceral adipose tissue, whereas leptin levels are more affected by subcutaneous adipose tissue.^5,6^

Several studies have shown that the PFAA profiles in obese people are altered compared with those in healthy people.^3,17^ There have been few reports regarding the variety of amino acid profiles in type 2 diabetics. In this study, the cluster analysis revealed that the amino acid profiles were different depending on the insulin level in type 2 diabetic patients, and higher plasma concentrations of Glu, Pro, BCAAs and Tyr were observed in the higher insulin group (Figure 1 and Table 2). Some of the interrelationships among the PFAAs, such as strong correlations among BCAAs,^18^ were conserved in the patients diagnosed with type 2 diabetes. In addition, these amino acids, which were elevated in the higher insulin group, were elevated in visceral obesity (visceral fat area⩾100 cm^2^) compared with the healthy controls.^3^ They showed good concordance with insulin-related variables, suggesting that the plasma insulin level has a critical role in regulating the concentrations of these plasma amino acids. Because a fasting plasma insulin level of 11μU ml^−1^ is reported to be one of the criteria for hyperinsulinemia in a study enrolling Japanese subjects,^19^ most of the subjects in the higher insulin group in this study were in a state of hyperinsulinemia. These results suggest the strong relationship among the accumulation of visceral fat, the plasma insulin level (insulin resistance), and the PFAA profiles in type 2 diabetic patients. Recently, Badoud *et al.*^20^ have also revealed that serum levels of BCAAs and Glu were strongly correlated with HOMA-IR by the study comparing the amino acid profiles among lean healthy subjects, metabolically healthy obese subjects and metabolically unhealthy obese subjects, which sustains our results.

Concerning the association between diabetes and the plasma amino acid concentrations, Leutscher^4^ was the first to report that patients with severe untreated diabetes mellitus had higher fasting plasma amino acid levels and that the administration of insulin caused a rapid return of their levels to normal. Many groups have since reported the changes in amino acid profiles in insulin resistant states, particularly the increase in BCAA concentrations.^21, 22, 23^ The reason for the BCAA increase in blood was thought to be a lower uptake of BCAAs into the muscles caused by decreased insulin action and decreased utilization of amino acids in muscles.^24^ A recent study revealed that BCAAs are metabolized in visceral adipose tissues and muscles, and insulin resistance causes the decrease in the expression levels of adipose-tissue BCAA catabolizing enzymes.^25,26^ Higher concentrations of Ala, Tyr, Glu and Pro were observed in the hyperinsulinemia group; however, the underlying mechanism of these changes is unclear. No amino acids showed a significant correlation with the blood glucose-related variables. In addition, few amino acids were correlated with 1,5-AG, which is a marker of short-time glycemic control,^27^ indicating that the glycemic change during several days might have little effect on the plasma amino acid profiles.

We revealed that plasma amino acids such as Glu, Ala, Trp and BCAAs were negatively correlated with adiponectin concentrations (Table 3). These strong correlations were also reflected in the result of our clustering analysis that BCAAs and Trp were categorized into the same group as adiponectin (Figure 1). Since Ala was highly correlated with BMI, it was categorized in the group of leptin and BMI. To our knowledge, this is the first report of the significant relationships between plasma amino acid profiles and adiponectin levels in human. Adiponectin is the most abundant secreted protein and is expressed exclusively in adipose tissue; it plays a pivotal role in the regulation of insulin sensitivity and metabolism. Many groups have reported that adiponectin concentration is decreased in obese people or diabetic patients and is strongly related to insulin resistance and hyperinsulinemia in humans.^7, 8, 9^ A recent study revealed that the injection of adiponectin into KKAy mice, which is a mouse model of obesity and/or type 2 diabetes, reversed insulin resistance, and dyslipidemia.^28^ Another group reported that changes in the plasma amino acid profile found in high-fat diet-induced mice were normalized by the injection of adiponectin.^29^ These results suggest that adiponectin levels might be the cause of insulin resistance or diabetes as well as likely factor for the metabolic shift that brings about changes in plasma amino acid profiles. However, the mechanism is still unknown.

We previously developed the ‘amino acid index' from different types of plasma amino acid concentrations for the discrimination of subjects with high visceral fat area (⩾100 cm^2^) by multivariate logistic regression analyses. The index showed a good correlation with visceral fat deposition independent of BMI or subcutaneous adipose tissue accumulation.^3^ In this study, we observed the significant correlations between amino acid indices evaluating visceral fat obesity and insulin-related variables or adiponectin concentration, indicating the strong association among visceral fat deposition, insulin resistance and plasma amino acid profile. These results did not show significant correlations with glycemic variables. However, HbA1c and FPG, which are used for the diagnosis of diabetes mellitus or the assessment of glycemic controls, were mainly correlated only with other glycemic variables such as 1,5-AG and GA, not with insulin-related variables or adiponectin. It is known that hyperinsulinemia or hypoadiponectinemia is the risk factor of coronary artery disease independent of fasting blood glucose.^30,31^ All of these data indicate that the indices determined from PFAA profiles may have the potential ability to assess the state of diabetic patients from the different viewpoint of the existing glycemic variables. To utilize these indices as monitoring markers, it is needed to study whether the normalization of the PFAA profile and the index values correlate with the improvement of insulin resistance or the increase in circulating adiponectin level.

Our study enrolling type 2 diabetic patients revealed that some amino acids and amino acid indices evaluating visceral fat obesity were strongly associated with insulin-related variables and plasma adiponectin levels, which are risk factors for developing cardiovascular diseases. This result stresses that PFAA profiles are hypothesized to be a useful marker for preventive diagnosis or healthcare assessment of diabetic patients. A prospective cohort study is needed for further investigation of whether changes in plasma amino acid concentrations occur prior to the changes in other metabolic variables such as insulin or adiponectin. Also, to investigate whether the index based on PFAA profiles can be used as an independent and early marker for health assessment in patients with type 2 diabetes, further studies of the prospective evaluation of the risk of developing future cardiovascular events in diabetic patients who had shown higher amino acid index values are needed.

## Figures and Tables

**Table 1:** Clinical variables according to the fasting plasma insulin and HbA1c levels

	*Total*	*Insulin*			*HbA1c*		
		High	*Low*	P*-value*	*High*	*Low*	P*-value*
		*male* N*=12 female* N*=14*	*male* N*=11 female* N*=14*		*male* N*=12 female* N*=14*	*male* N*=11 female* N*=14*	
*Insulin (μU ml^−1^)*							
M	10.1±7.8	**14.9±8.2**	**4.8±1.2**	0.0006***	9.7**±**7.7	10.5**±**8.2	0.8061
F	8.6**±**3.4	**11.3±1.9**	**5.8±2.0**	<0.0001***	8.8**±**2.9	8.4**±**4.0	0.7505
							
*HbA1c (%)*							
M	7.4**±**1.2	7.5**±**1.3	7.3**±**1.1	0.7389	**8.4±0.7**	**6.4±0.7**	<0.0001***
F	7.2**±**0.6	7.2**±**0.7	7.1**±**0.6	0.8383	**7.7±0.4**	**6.7±0.3**	<0.0001***
							
*Age (years)*							
M	52.3**±**10.3	50.2**±**7.9	54.7**±**12.4	0.3006	48.8**±**11.2	56.3**±**8.0	0.0804
F	55.8**±**11.5	54.6**±**13.4	56.9**±**9.7	0.6203	55.9**±**10.6	55.6**±**12.8	0.9365
							
*BMI (kg m^−^^2^*)							
M	27.0**±**4.9	28.3**±**5.4	25.6**±**4.0	0.1970	27.0**±**6.0	27.0**±**3.6	0.9804
F	26.3**±**4.6	**28.1±5.3**	**24.5±3.0**	0.0315*	26.1**±**3.1	26.5**±**5.9	0.8055
							
*FPG (mg dl^−1^)*							
M	150.5**±**36.5	158.7**±**44.6	141.5**±**23.9	0.2707	**168.2±34.6**	**131.2±28.8**	0.0114*
F	143.6**±**32.2	144.6**±**30.6	142.6**±**34.8	0.8774	**160.6±34.1**	**126.6±19.0**	0.0031**
							
*GA (%)*							
M	20.0**±**4.4	19.7**±**4.8	20.3**±**4.2	0.7362	**22.2±4.3**	**17.6±3.3**	0.0093**
F	19.8**±**2.8	19.5**±**2.6	20.1**±**3.0	0.5839	**21.4±2.6**	**18.1±1.9**	0.0006***
							
*1,5-AG (μg ml^−1^)*							
M	7.5**±**4.7	7.4**±**5.1	7.6**±**4.6	0.9188	**4.6±2.7**	**10.7±4.5**	0.0008***
F	7.7**±**4.6	7.5**±**5.1	7.8**±**4.4	0.8712	**5.2±4.6**	**10.1±3.3**	0.0032**
							
*HDL cholesterol (mg dl^−1^)*							
M	51.8**±**9.7	49.6**±**10.2	54.2**±**9.0	0.2675	51.5**±**10.3	52.1**±**9.6	0.8884
F	58.0**±**11.6	**53.5±9.1**	**62.5±12.5**	0.0379*	60.1**±**12.0	55.9**±**11.2	0.3387
							
*LDL cholesterol (mg dl^−1^)*							
M	128.3**±**31.7	132.4**±**29.8	123.3**±**34.8	0.5155	125.8**±**40.4	130.7**±**21.7	0.7261
F	137.4**±**27.9	142.9**±**24.5	131.8**±**30.8	0.2991	137.8**±**29.4	136.9**±**27.4	0.9370
							
*Triglyceride (mg dl^−1^)*							
M	184.4**±**100.4	212.5**±**122.2	153.7**±**61.4	0.1658	190.2**±**125.7	178.1**±**68.7	0.7807
F	115.1**±**49.4	**135.6±35.8**	**94.6±53.7**	0.0252*	116.9**±**43.3	113.3**±**56.4	0.8495
							
*C-peptide (ng ml^−1^)*							
M	2.2**±**0.8	**2.7±0.7**	**1.7±0.3**	0.0003***	2.3**±**0.9	2.1**±**0.7	0.6231
F	1.8**±**0.5	**2.2±0.3**	**1.5±0.4**	<0.0001***	1.9**±**0.3	1.8**±**0.6	0.4412
							
*HOMA-IR*							
M	3.6**±**2.6	**5.4±2.5**	**1.7±0.5**	<0.0001***	3.9**±**2.5	3.3**±**2.8	0.5985
F	3.0**±**1.4	**4.1±1.2**	**2.0±0.8**	<0.0001***	3.4**±**1.4	2.6**±**1.4	0.1437
							
*Leptin (ng ml^−1^)*							
M	7.8**±**4.7	9.6**±**5.4	5.8**±**2.7	0.0501	7.2**±**4.5	8.4**±**4.9	0.5259
F	14.4**±**9.4	**18.9±10.9**	**10.0±4.5**	0.0093**	14.3**±**7.7	14.5**±**11.1	0.9593
							
*Adiponectin (μg ml^−1^)*							
M	6.3**±**2.2	**5.3±1.5**	**7.3±2.4**	0.0255*	5.6**±**1.3	7.0**±**2.7	0.1146
F	6.9**±**2.6	**5.4±2.1**	**8.3±2.3**	0.0020**	6.2**±**2.1	7.5**±**2.9	0.1809
							
*Antidiabetic drugs (taken/not taken)*^†^							
M	15/8	8/4	7/4	0.8789	8/4	7/4	0.8789
F	21/7	11/3	10/4	0.6625	**13/1**	**8/6**	0.0291*

Abbreviations: BMI, body mass index; F, female; FPG, fasting plasma glucose; GA, glycoalbumin; HbA1c, hemoglobin A1c; HDL, high-density lipoprotein; HOMA-IR, homeostasis model assessment of insulin resistance; LDL, low-density lipoprotein; M, male.

Data are expressed as the mean±s.d. *P*-values were determined by Student's *t*-test or *χ*^2^ test (^†^) between high and low groups of fasting plasma insulin concentrations or HbA1c. Significant differences are highlighted in bold letters and shown as *P<0.05, **P<0.01 and ***P<0.001.

**Table 2:** Plasma amino acid concentrations according to the levels of fasting plasma insulin and HbA1c

*Group*	*Total*	*Insulin*			*HbA1c*		
		*High*	*Low*	P*-value*	*High*	*Low*	P*-value*
		*male* N*=12* *female* N*=14*	*male* N*=11* *female* N*=14*		*male* N*=12* *female* N*=14*	*male* N*=11* *female* N*=14*	
*Essential amino acids (μmol l^−1^)*							
Val							
M	281.7±33.4	293.3±24.7	269.0±38.1	0.0822	289.2±27.5	273.5±38.6	0.2724
F	249.3±40.6	**268.5**±**40.5**	**230.1**±**31.3**	0.0093**	263.3±47.8	235.3±26.7	0.0666
Ile							
M	84.0±15.6	87.4±15.8	80.3±15.3	0.2862	85.0±14.9	83.0±17.1	0.7661
F	67.0±12.1	**73.7**±**9.9**	**60.4**±**10.5**	0.0019**	**71.9**±**12.5**	**62.2**±**9.8**	0.0302*
Leu							
M	156.5±19.4	162.4±14.5	150.1±22.7	0.1351	157.9±16.1	155.0±23.3	0.7347
F	130.8±19.2	**137.9**±**18.2**	**123.7**±**18.1**	0.0483*	**138.2**±**18.9**	**123.4**±**17.0**	0.0391*
His							
M	86.4±6.9	86.2±5.6	86.7±8.3	0.8828	84.1±6.3	89.0±6.7	0.0812
F	79.3±12.8	80.0±10.4	78.5±15.1	0.7596	80.1±10.8	78.4±14.9	0.7293
Phe							
M	64.7±9.2	65.6±9.1	63.8±9.8	0.6539	63.7±8.5	65.8±10.3	0.5979
F	60.5±6.6	62.2±6.7	58.8±6.3	0.1779	60.3±5.0	60.7±8.1	0.8632
Trp							
M	59.8±7.7	62.5±7.9	56.9±6.7	0.0869	60.2±7.3	59.4±8.6	0.7989
F	51.6±7.8	54.1±6.6	49.2±8.3	0.0953	52.9±7.5	50.4±8.1	0.4049
Met							
M	29.5±8.7	31.9±10.7	26.9±5.2	0.1756	29.8±11.4	29.2±4.9	0.8751
F	24.2±4.1	**25.7**±**2.7**	**22.6**±**4.7**	0.0390*	25.2±3.4	23.1±4.6	0.1876
Thr							
M	122.6±21.9	129.0±25.4	115.7±15.6	0.1512	118.8±21.1	126.8±23.0	0.3983
F	114.7±27.0	116.7±17.8	112.6±34.4	0.6938	119.0±28.3	110.3±25.9	0.4030
Lys							
M	206.7±29.7	211.7±30.4	201.3±29.4	0.4153	201.4±32.0	212.6±27.4	0.3790
F	199.6±38.6	211.4±41.5	187.9±32.9	0.1096	209.5±34.7	189.8±41.1	0.1831
*Non-essential amino acids (μmol l^−1^)*							
Ser							
M	104.4±19.0	109.5±18.3	98.9±19.0	0.1873	101.6±20.2	107.6±18.0	0.4595
F	105.7±22.2	98.4±17.2	113.1±24.7	0.0789	107.1±17.9	104.4±26.4	0.7518
Gly							
M	194.5±31.4	189.1±23.3	200.4±38.6	0.4031	190.5±35.8	198.9±26.7	0.5326
F	189.7±50.7	186.1±34.1	193.2±64.5	0.7213	188.3±35.6	191.0±63.8	0.8919
Ala							
M	447.4±80.9	473.1±83.2	419.5±71.8	0.1149	449.4±91.4	445.4±72.2	0.9087
F	422.5±83.8	**462.6**±**81.9**	**382.3**±**66.3**	0.0084**	445.8±65.0	399.1±95.8	0.1439
Tyr							
M	73.3±15.7	**80.9**±**17.1**	**65.1**±**8.7**	0.0118*	76.2±20.3	70.2±8.1	0.3703
F	63.5±10.2	**68.2**±**7.6**	**58.8**±**10.5**	0.0115*	63.2±10.2	63.8±10.5	0.8732
Glu							
M	43.9±16.1	**51.5**±**18.7**	**35.7**±**6.6**	0.0151*	42.4±9.3	45.6±21.7	0.6439
F	32.9±15.3	33.8±17.6	30.5±9.8	0.5477	34.4±18.1	30.0±8.7	0.4169
Pro							
M	181.2±50.6	**203.0**±**58.2**	**157.4**±**26.8**	0.0271*	192.4±57.6	169.0±41.0	0.2795
F	157.3±60.5	171.7±49.2	142.9±68.8	0.2128	174.9±70.9	139.7±43.6	0.1256
Gln							
M	584.0±60.0	569.5±64.8	599.8±52.7	0.2344	579.6±61.1	588.7±61.4	0.7235
F	565.0±74.0	558.6±57.1	571.4±89.5	0.6553	570.7±66.4	559.3±83.0	0.6912
Cit							
M	29.6±7.7	27.9±7.7	31.4±7.7	0.2934	26.9±6.5	32.5±8.2	0.0828
F	26.5±9.9	25.4±10.0	27.5±10.0	0.5711	26.9±9.4	26.0±10.6	0.8043
Arg							
M	99.4±15.6	101.0±14.8	97.7±17.0	0.6257	99.0±17.8	99.9±13.7	0.8984
F	85.2±19.0	83.3±16.6	87.1±21.6	0.6061	83.7±18.7	86.6±19.9	0.6994
Orn							
M	50.6±12.3	52.9±14.2	48.1±9.8	0.3627	49.7±15.8	51.6±7.5	0.7083
F	48.8±10.4	49.6±11.5	48.1±9.4	0.7086	51.9±10.3	45.7±9.8	0.1162
Asn							
M	47.9±5.5	48.8±5.9	46.9±5.0	0.4121	45.8±4.3	50.1±5.9	0.0596
F	43.8±7.2	44.8±7.8	42.7±6.6	0.4612	44.8±5.5	42.8±8.6	0.4714
a-ABA							
M	22.3±4.8	21.8±5.2	22.9±4.4	0.6019	22.2±6.3	22.5±2.6	0.8921
F	21.7±6.7	20.8±5.7	22.6±7.7	0.4879	23.0±4.8	20.5±8.2	0.3231
Amino acid index-1							
M	1.27±1.07	**1.88**±**0.85**	**0.59**±**0.87**	0.0018**	1.34±1.00	1.18±1.18	0.7267
F	0.35±1.07	**0.79**±**1.09**	**−0.09**±**0.88**	0.0261*	0.63±1.14	0.07±0.95	0.1678
Amino acid index-2							
M	17.0±3.4	**18.6**±**2.7**	**15.2**±**3.3**	0.0117*	18.0±3.6	15.8±2.9	0.1185
F	15.5±3.7	**17.3**±**3.3**	**13.6**±**3.2**	0.0065**	16.2±3.9	14.7±3.6	0.2958

Abbreviations: F, female; HbA1c, hemoglobin A1c; M, male.

Data are expressed as the mean±s.d. *P*-values were determined by Student's t-test between high and low groups of fasting plasma insulin or HbA1c levels. Significant differences are highlighted in bold letters and shown as *P<0.05, **P<0.01 and ***P<0.001.

**Table 3:** Pearson's correlation coefficients among plasma amino acid concentrations and clinical variables

	*BMI*	*FPG*	*HbA1c*	*GA*	*1,5-AG*	*C-peptide*	*Insulin*	*HOMA-IR*	*Leptin*	*Adiponectin*
*Coefficient of correlation*										
Glucose related variables										
* *FPG	−0.133	—	0.715***	0.778***	−0.629***	0.154	−0.094	0.321*	−0.126	−0.017
* * HbA1c	−0.06	0.715***	—	0.761***	−0.758***	0.088	−0.154	0.133	−0.121	−0.176
										
*Amino acids*										
Essential amino acids										
* *Val	0.109	0.221	0.401**	0.104	−0.240	0.275	0.228	0.309*	−0.084	−0.535***
* *Ile	0.153	0.259	0.272	0.165	−0.236	0.280*	0.189	0.282*	−0.054	−0.534***
* *Leu	0.128	0.251	0.289*	0.116	−0.163	0.238	0.252	0.330*	−0.137	−0.468***
* *His	0.121	−0.091	−0.042	−0.089	0.058	0.049	0.020	0.013	−0.059	−0.027
* *Phe	0.188	−0.234	−0.109	−0.294*	0.214	0.086	0.213	0.062	−0.021	−0.188
* *Trp	0.189	−0.181	0.049	−0.212	0.122	0.270	0.291*	0.182	0.060	−0.440***
* *Met	0.024	−0.038	0.190	−0.085	−0.099	0.224	0.197	0.153	−0.097	−0.172
* *Thr	0.041	−0.283*	−0.045	−0.226	0.039	−0.004	0.096	−0.051	−0.042	−0.190
* *Lys	−0.006	−0.277*	0.026	−0.248	0.090	0.073	0.161	0.035	−0.052	−0.205
Non-essential amino acids										
* *Ser	−0.113	−0.289*	−0.079	−0.187	0.196	−0.146	0.046	−0.09	−0.145	0.221
* *Gly	−0.137	−0.218	−0.162	−0.161	0.129	−0.157	−0.150	−0.219	−0.145	0.062
* *Ala	0.376**	0.111	0.179	−0.050	−0.141	0.318*	0.306*	0.286*	0.254	−0.524***
* *Tyr	0.285*	−0.019	0.195	−0.065	−0.048	0.426**	0.425**	0.386**	0.114	−0.248
* *Glu	0.252	0.060	0.055	−0.044	−0.043	0.519***	0.564***	0.506***	0.066	−0.381**
* *Pro	0.301*	0.243	0.325*	0.132	−0.333*	0.317*	0.237	0.337*	0.279*	−0.248
* *Gln	0.039	−0.336*	−0.074	−0.168	0.048	−0.112	−0.060	−0.194	−0.047	0.028
* *Cit	−0.259	−0.064	−0.055	0.127	0.085	−0.154	−0.252	−0.236	−0.142	0.220
* *Arg	−0.033	−0.194	−0.028	−0.149	0.103	0.082	0.081	0.019	−0.087	0.082
* *Orn	−0.056	−0.153	0.033	−0.113	0.110	0.119	0.101	0.041	0.047	0.030
* *Asn	0.151	−0.266	−0.111	−0.253	0.129	0.006	0.099	−0.043	−0.018	−0.146
* *a-ABA	−0.129	−0.025	0.165	−0.003	−0.031	−0.093	−0.053	−0.095	−0.202	−0.224
Amino acid index-1	0.342*	0.229	0.280*	0.071	−0.203	0.546***	0.547***	0.569***	0.116	−0.523***
Amino acid index-2	0.304*	0.324*	0.421**	0.164	−0.298*	0.447**	0.387**	0.483***	0.162	−0.468***

Abbreviations: BMI, body mass index; FPG, fasting plasma glucose; GA, glycoalbumin; HbA1c, hemoglobin A1c; HOMA-IR, homeostasis model assessment of insulin resistance.

Pearson's product-moment correlation coefficients were used to evaluate the correlations between plasma amino acid and glucose- or insulin-related variables. Significant correlations are highlighted in bold letters and shown as *P<0.05, **P<0.01 and ***P<0.001.

## Notes

HN, HJ, KN, YN, NS and HM are employees of Ajinomoto Co., Inc. TW is the CEO of Link and Communication Co., Inc. TW and KI received research grants from Ajinomoto Co., Inc. No other potential conflicts of interest relevant to this article are declared.
